# Development of Temporal Model for Forecasting of *Helicoverpa armigera* (Noctuidae: Lepidopetra) Using Arima and Artificial Neural Networks

**DOI:** 10.1093/jisesa/ieac019

**Published:** 2022-05-05

**Authors:** Ramana Narava, Sai Ram Kumar D V, Jagdish Jaba, Anil Kumar P, Ranga Rao G V, Srinivasa Rao V, Suraj Prashad Mishra, Vinod Kukanur

**Affiliations:** 1 Department of Entomology, Agricultural College, Acharya N.G. Ranga Agricultural University, Bapatla, 522101, Guntur, Andhra Pradesh, India; 2 International Crops Research Institute for the Semi-Arid Tropics (ICRISAT), Patancheru, 502324, Telangana, India; 3 Department of Plant Pathology, Agricultural College, Acharya N.G. Ranga Agricultural University, Bapatla, 522101, Guntur, Andhra Pradesh, India; 4 Department of Statistics and Computer applications, Agricultural College, Acharya N.G. Ranga Agricultural University, Bapatla, 522101, Guntur, Andhra Pradesh, India

**Keywords:** *H. armigera*, forecast, ARIMA, Artificial Neural Networks

## Abstract

*Helicoverpa armigera* (Hübner) (Noctuidae: Lepidopetra) is a polyphagous pest of major crops grown in India. To prevent the damage caused by *H*. *armigera* farmers rely heavily on insecticides of diverse groups on a regular basis which is not a benign practice, environmentally and economically. To provide more efficient and accurate information on timely application of insecticides, this research was aimed to develop a forecast model to predict population dynamics of pod borer using Autoregressive Integrated Moving Average (ARIMA) and Artificial Neural Networks (ANN). The data used in this study were collected from the randomly installed sex pheromone traps at International Crops Research Institute for the Semi-arid Tropics (ICRISAT), Patancheru, Hyderabad. Several ARIMA (p, d, q) (P, D, Q) and ANN models were developed using the historical trap catch data. ARIMA model (1,0,1), (1,0,2) with minimal BIC, RMSE, MAPE, MAE, and MASE values and higher R^2^ value (0.53) was selected as the best ARIMA fit model, and neural network (7-30-1) was found to be the best fit to predict the catches of male moths of pod borer from September 2021 to August 2023. A comparative analysis performed between the ARIMA and ANN, shows that the ANN based on feed forward neural networks is best suited for effective pest prediction. With the developed ARIMA model, it would be easier to predict *H*. *armigera* adult population dynamics round the year and timely intervention of control measures can be followed by appropriate decision-making schedule for insecticide application.

*Helicoverpa armigera* (Hübner) (Lepidoptera: Noctuidae) synonymously known by many common names (e.g., pod borer, tobacco bud worm, tomato fruit borer, bollworm, head caterpillar, and American bollworm) is considered as one of the world’s most important pest of both agricultural and horticultural crops. It is considered as obnoxious polyphagous pest in Indian sub-continent that causes heavy economic losses in many crops (Armes et al. 1996). In many cropping systems it easily attains a major pest status due to its physiological, ethological, and ecological characteristics viz., polyphagy, wide geographical range, migratory potential, facultative diapause, and high fecundity (Fitt 1989). It is also considered as most difficult pest to control because of its recidivist nature of developing resistance to almost all the insecticides deployed for its control (Forrester et al. 1993, Kranthi et al. 2001). In many cropping ecosystems apart from causing heavy economic losses, it has also led to serious socio-ecological problems. In India, *H. armigera* has been recorded on at least 181 plant species from 45 families, more particularly in field crops such as cotton, pigeonpea, chickpea, groundnut, sorghum, and vegetable crops (Manjunath et al. 1989). Indian agriculture is diverse and is characterized by small land holdings with diversified agricultural practices. Cropping patterns in India typically ensure the presence of five to six different host crops in varying proportions at any given time of the growing season (Manjunath et al. 1989), resulting in a heterogeneous matrix of hosts that provide ideal habitat for *H. armigera* to move between hosts and geographic areas throughout the year. In addition, the presence of three primary cropping scenarios in India is determined by the monsoon pattern (Singh and Bain 1986) (i.e., southwest monsoons: June to September and northeast monsoons: October to December), allowing the population to migrate across the subcontinent. 

Management of *H. armigera* relies heavily on insecticides. Exclusion of other methods of management and indiscriminate use of insecticides has resulted in the development of resistance and resurgence of the pest (Phokela et al. 1990, Sreekanth et al. 2016). Integrated pest management (IPM) is the most accomplished way for pod borer management. However, availability of alternative hosts, topography, farming practices, changes in population dynamics, and climate change largely hampers the success of IPM practices. Climatic seasonality, availability of crop hosts, management practices, other inter species interactions, and ecological synchrony are the determinants of the insect–pest infestation. In order to understand the adult population dynamics of *H. armigera*, an annual pattern of male moths has been monitored using sex pheromone traps at several experimental sites at the International Crops Research Institute for the Semi-Arid Tropics (ICRISAT), Patancheru, since 1977 (Pawar et al. 1988). The phenology details of the *H. armigera* provide the basic information about the underlying mechanisms that regulate the seasonal occurrence and relationship between the level of insect damage and adult trap catches.

In IPM, pheromone trap catches monitoring has been successfully used to administer the need-based sprays of insecticides to avoid pest attaining economic threshold levels (Witzgall et al. 2010). Knowledge of crop phenology and insect appearance, as well as moth population monitoring, will aid in regulating pest populations below the economic threshold level (ETL), and allowing the prediction of pest appearance timing at each crop developmental stage, as well as seasonal and temporal population dynamics to continuously monitor subsistence insect–pest management. Certainly, seasonal forecasting of insect–pest pressure is the key for effective management of any insect pest. The weather is also one of the major factors responsible for infestation of any insect pest. The major weather variables viz., temperature, rainfall, and relative humidity significantly influence the pest populations (Siswanto et al. 2008) including *H. armigera* (Jaba et al. 2017).

A prediction model that is based on the sex pheromone trap catch data was developed in the current research. The accuracy of prediction models built using weather data is not more than 60%. However, the models that are built on insect activity as a predictor, have resulted in more accurate prediction. Thus, we attempted to use Auto Regressive Integrated Moving Average (ARIMA) and Artificial Neural Networks (ANN) for prediction without considering any exogenous parameters. The current prediction models are capable of properly predicting moth activity as well as pest population dynamics over time. It can be a significant scientific tool for forewarning the advent of pest and timely intervention of management measures before damage occurs. Nevertheless, few concerted efforts have been made so far to develop a forecasting model for insect pest seasonal occurrence. Most of the earlier studies have used regression models (both linear and nonlinear) for pest and disease forecasting models (Agrawal and Mehta 2007).

Long-term forecast models of pest pressure are vital for the effective management of many agricultural insect pests. Crop modelling can act as a decision-making support system for concurrent climate scenarios. In this study we made an attempt to model the seasonal occurrence of *H. armigera* using the pheromone trap catches data collected at ICRISAT, Patancheru, India.

## Material and Methods

### Study Site and Weather

Present study was carried out at the International Crops Research Institute for the Semi-Arid Tropics (ICRISAT), Patancheru (17.51 °N, 78.27 °E, and 545 m), Hyderabad, Telangana, India. The area receives an annual mean rainfall greater than 750 mm, with main rainy season between June and September. The study area has mosaic landscape and suitable to grow most of semi-arid tropics crops, however at ICRISAT crops like chickpea, groundnut, pigeonpea, sorghum, pearl millet, and finger millet are grown.

### Trap Catches of *H. armigera*

The incidence of *H*. *armigera* on various ICRISAT mandate crops is being monitored from the last twenty-five years. However, in the present study, the pheromone trap data of last five years (2015–2021) was used for building ARIMA and ANN models. Around 10–12 pheromone traps (Pest Control India (PCI) Pvt Ltd, Bangalore, India) were installed in different locations of ICRISAT at 1.5 m height above the crop canopy. Pheromone lures comprised a polyethylene vial containing 2 mg of Z-11-Hexadecenal, and Z-9-Hexadecenal, placed in the centre of the trap. Pheromone lures were replaced with new ones at every 30 d intervals. The trapping of male moths was continued across the years 2015–2021 (up to August), irrespective of the crops grown at ICRISAT. Numbers of *H. armigera* catches were recorded at weekly intervals and expressed as mean number of male moths/trap/week. This dataset was used to develop the forecast models. The modelling procedure was performed as follows. The data were visualised to comprehend the *H. armigera* population dynamics, distribution, and onset of the economic injury levels at critical crop growth stages.

### ARIMA Model

Autoregressive Integrated Moving Average (ARIMA) is a class of statistical models for analysing and forecasting time series data in order to obtain future prediction from historical data. It explicitly caters to a suite of standard structures in time series data, and as such provides a simple yet powerful method for making skilful time series forecasts. In theory, ARIMA includes three components: auto-regression (AR), moving-average (MA), and integration (I) terms.

### The Box–Jenkins Methodology

Box–Jenkins analysis refers to a systematic method of identifying, fitting, checking, and using integrated autoregressive, moving average (ARIMA) time series models. The ARIMA models are capable of modelling both nonseasonal (p, d, q) as well as a wide range of seasonal data (P, D, Q). ARIMA shows that there is a relation between present value and past value and residuals respectively. In this study, Box–Jenkin’s methodology was applied for identifying the best ARIMA models and residuals using the time series data. The multiplicative seasonal ARIMA model is represented as follows (1)


$$\begin{array}{l} \Phi_{P}\left(B^{s}\right)\varphi p\;\left(B\right){\nabla^{D}}_{s}{\nabla^{d}}_{z\;t}=\theta q\;\left(B\right)\Theta Q\;\left(B^{s}\right)\;a_{t} \end{array}$$ (1)

Where

-
$\Phi_{P}\left(B^{s}\right)\;=\;1\;-\Phi 1B^{s}-...\;-\Phi_{P}B^{sP}$ is the seasonal AR operator of order P;
-
$\Phi p\;=\;1\;-\varphi 1B\;-...\;-\varphi_{p}B^{P}$ is the regular AR operator of order p;
-
$\nabla^{D}s\;=\;{\left(1\;-\;B^{s}\right)}^{D}$ represents the seasonal differences and ∇d = (1 − B)d the regular differences;
-
$\Theta Q\;\left(B^{s}\right)\;=\;1\;-\Theta 1B^{s}-...\;\Theta Q\;B^{s}$ Q is the seasonal moving average operator of order Q;
-
$\theta_{q}\left(B\right)\;=\;\left(1\;-\theta_{1}B\;-...\;-\theta_{q}B_{q}\right)$ is the regular moving average operator of order q;
- a_t_ is a white noise process

The p, q, d values of ARIMA can be computed automatically by using Auto-ARIMA function a variant of ARIMA. Auto-ARIMA iteratively enumerates the information criteria used to select the best *p*, *q*, *d* values such as Akaike Information Criterion (AIC), Bayesian Information Criterion (BIC), Hannan-Quinn Information Criterion (HQIC), Schwarz Criterion (SC), and Out of Bag (OOB). Among the different criteria, AIC was used in this work for optimizing best fit using the following equation (2):


$$\begin{array}{l} AIC\;=\;-2\;log\;\left(Maximum\;likelihood\right)\;+\;2k \end{array}$$ (2)

where *k* = *p* + *q* + 1 if the model contains an intercept or constant term and *k* = *p* + *q* otherwise. The best *p*, *q*, *d* values were determined based on the lowest AIC values found under different values of *p*, *q*, and *d* (Cryer 2008).

In this study to shortlist the best fit ARIMA model among the several combinations performed, the models with relatively small AIC, high R-Square, and low MAPE values, were selected. A correlogram with no significant pattern by correlation function (ACF) and partially auto correlation function (PACF) was used to model the predictions.

### ADF and KPSS Tests for Stationary Testing

The input data must be stationary and homogeneous before fitting the ARIMA model. This is because the mean and variance of a stationary data is constant over time, which can help in easier prediction. Our data was tested with ADF (Augmented Dickey-Fuller) test (α = 0.05) for stationarity. The ADF test statistic is an estimated coefficient from the method of least squares regression formula (3). If the *P*-value > α, condition of the ADF test is met, the null hypothesis cannot be rejected which means the data is stationary (Cheung and Lai 1995). The KPSS (Kwiatkowski–Phillips–Schmidt–Shin), is a type of unit root test that tests for the stationarity of a given series around a deterministic trend. It breaks up a series into three parts: a linear regression deterministic trend (βt), a (random walkrt), and a stationary error (ε_t_), with the regression equation (4) (Kokoszka and Young 2016).


$$\begin{array}{l} \Delta \lambda_{t}=\;\;\;\alpha_{0\;\;\;}+\alpha_{2}t+\underset{i=1}{\overset{k}{\sum }}\;\beta \Delta \lambda_{t-1}+\varepsilon_{t} \end{array}$$ (3)

Where $\lambda_{t}$ denotes the weekly index of the individual stock at time *t*, β is the coefficient to be estimated, *k* is the number of lagged terms, t is the trend term, $\alpha_{2}$ is the estimated coefficient for the trend, $\alpha_{0\;}$ is the constant, and ε is the white noise.


$$\begin{array}{l} x_{t}\;=\;r_{t}\;+\;\beta t\;+\;\varepsilon_{1} \end{array}$$ (4)

### Artificial Neural Network (ANN) Model

Neural Networks are data-driven, self-adaptive, nonparametric statistical methods which mimic the human brain. The main advantage of a neural network is its ability to model complex nonlinear relationship without a prior assumption of the nature of the relationship. The ANN model performs a nonlinear functional mapping from the past observations $\left(y_{t-1},y_{t-2},.,y_{t-p}\right)$ to the future value $y_{t}$, i.e.,


$$\begin{array}{l} y_{t}=f\left(y_{t-1},\;y_{t-2},\;\ldots .,\;y_{t-p},\;w\right)+\varepsilon_{t} \end{array}$$

where w is a vector of all parameters and f is a function determined by the network structure and connection weights. The important task of the ANN modelling for a time series is to choose an appropriate number of hidden nodes (*k*) as well as the dimensions of the input vector p (the lagged observations). The ANN model was employed as outlined by Areef and Radha (2020).

A multilayer feed forward neural network was fitted to the data with the help of *nnetar* package, which is extensively used for fitting univariate time series. According to the AIC, the optimal number of seasonal (p) or nonseasonal (P) lags were used as inputs. As a result, the fitted model is called an NNAR (p, P, k) [m] model, which is analogous to an ARIMA (p,0,0) (P,0,0) [m] model but with nonlinear functions.

### Forecast Evaluation of the Models

The forecasting ability of different models is assessed with respect to common performance measures, viz. root mean squared error (RMSE), mean absolute error (MAE), and mean absolute percentage error (MAPE).


$$\begin{array}{l} RMSE:\;RMSE=\;\sqrt{\frac{\overset{T}{\underset{t=1}{\sum }}{\left(y_{t}-{\hat{y}}_{t}\right)}^{2}}{T}} \end{array}$$

$$\begin{array}{l} MAPE:MAPE=\left[\underset{t=1}{\overset{n}{\sum }}\;\left|\frac{y_{t}-{\hat{y}}_{t}}{y_{t}}\right|\;\times 100\right]/n \end{array}$$

Where, $y_{t}$ = actual moth count, ${\hat{y}}_{t}$ = predicted moth count, *T* = sample size

## Results

### Data Selection and Curation for ARIMA

We used adult male population catches as a real univariate time series data to determine the necessary input for forecasting the *H. armigera* incidence. For validating the selected model, the normality of the residuals was tested. Normality testing of the dataset was done by simple normal distribution and Q–Q plots. In the current study, we started with the initial preprocessing of the data to make it stationary by performing ADF and KPSS tests and the results are presented in Table 1; where the *P* values were lower than 0.05 i.e., 0.01 and 0.01 for both the tests, respectively, which confirmed the data was stationary.

**Table 1.** Augmented Dickey–Fuller (ADF) and Kwiatkowski–Phillips–Schmidt–Shin (KPSS) test for data stationary testing

Stationarity test	Critical value	*P*-value
ADF test	−9.4416	0.01*
KPSS test	2.1577	0.01*

*Significant at 0.05 level of significance.

### Fitting of ARIMA Model

**Fig. 1.**
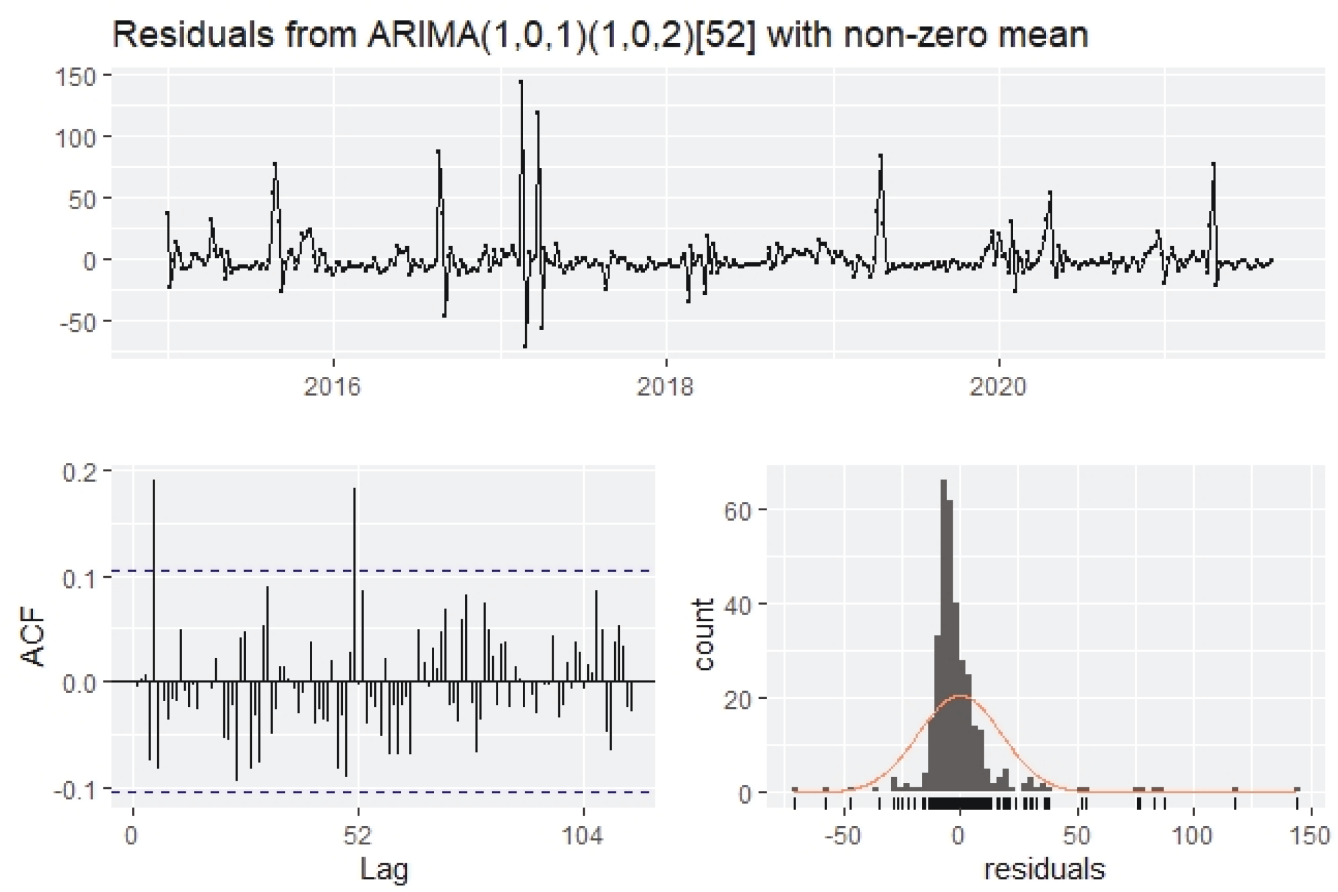
Residual plot of ARIMA model for *H. armigera* moth catches.

The time series was evidently nonstationary, but it became stationary at the first difference, as confirmed by the ADF test because the calculated values were less than critical values. The ARIMA models for the predicted *H. armigera* populations are shown in Table 2. Out of the seven developed ARIMA models, the best-fit model for the *H. armigera* trap catches was ARIMA (1,0,1), (1,0,2) where the R^2^ value was higher (0.53) with root mean square error, absolute mean error, mean absolute scaled error, mean absolute percentile error, and Bayesian information criterion values as 17.74, 9.42, 0.99, 93.70, and 3002.56, respectively. The model parameters for the best fit ARIMA (1,0,1) (1,0,2) are presented in Table 3. The *P*-value of the Ljung–Box test for *H. armigera* moth catches was 4.5 (>0.05), indicating the independence of residuals; Fig. 1 illustrates the residuals of the selected model.

**Table 2.** The tentative models of ARIMA (p d q) (P D Q) with values of model selection indices

S. No.	ARIMA (p d q) (P D Q) model	R^2^	RMSE	MAE	MAPE	MASE	BIC
1.	ARIMA (1,0,0) (1,0,1)	0.33	34.11	17.47	114.33	1.04	4850.18
2.	ARIMA (1,0,0) (1,0,2)	0.48	32.45	16.82	94.15	1.00	3016.61
3.	ARIMA (1,0,1) (1,0, 2)	0.53	17.74	9.42	93.70	0.99	3002.56
4.	ARIMA (2,0,0) (1,0,2)	0.33	34.89	16.74	110.48	1.00	4758.62
5.	ARIMA (1,0,0) (1,0,0)	0.50	33.29	17.18	113.39	1.02	4362.94
6.	ARIMA (0,0,1) (1,0,2)	0.45	34.92	16.66	96.22	1.00	3984.23
7.	ARIMA (0,0,1) (0,0,1)	0.50	33.45	16.73	96.20	1.02	3874.38

**Table 3.** Model parameters of the best fit, ARIMA (1,0,1) (1, 0, 2)

Model	Coefficients		Estimate	SE±
ARIMA (1,0,1) (1, 0, 2)	AR	Lag 1	0.3949	0.1388
	MA	Lag 1	0.1247	0.1465
	AR, Seasonal	Lag 1	0.3162	1.8439
	MA, Seasonal	Lag 1	-0.1094	1.8429
	MA, Seasonal	Lag 2	0.0698	0.4197

### Fitting of ANN Model

**Fig. 2.**
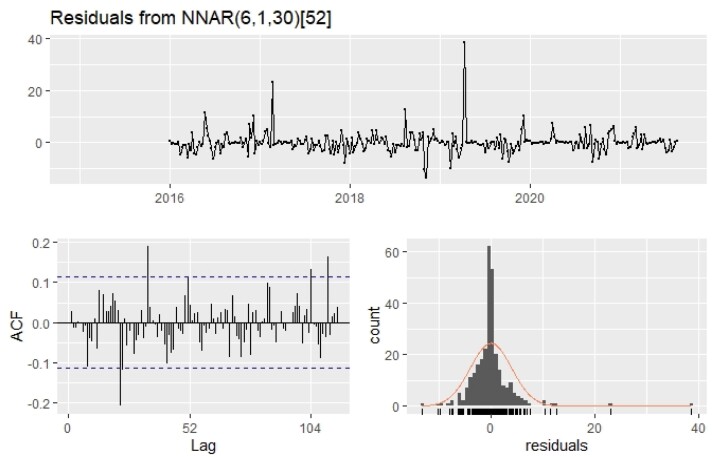
Residual plot of ANN model for *H. armigera* moth catches.

A multilayer feedforward network architect with backpropagation was considered for fitting and modelling old world bollworm, *H. armigera* moth catch series. As a result, 18 lags were identified as optimal for network input nodes. Various network topologies were trained by increasing the number of hidden nodes from 4 to 35 and using the sigmoid as an activation function in the hidden layer. Among several models, the 10 best performing models are listed in Table 4, based on the lowest of RMSE, MAE, and MASE values. A neural network 7-30-1 (7 input nodes, 30 hidden nodes, and 1 output) outperformed all other neural networks with lowest RMSE (3.928), MAE (2.145), MAPE (26.767), and MASE (0.169) values. The *P*-value of the Ljung–Box test for pod borer moth catches was 0.35 (>0.05), indicating the independence of residuals; Fig. 2 illustrates the residuals of the selected model.

**Table 4.** Performance of artificial neural network (ANN) models with their model selection criteria values

Network structure	MAPE	RMSE	MAE	MASE
7-4-1	59.335	9.984	5.337	0.420
7-5-1	55.915	8.852	4.899	0.386
7-6-1	52.507	7.913	4.547	0.358
7-12-1	38.478	6.011	3.358	0.264
7-13-1	37.407	6.046	3.230	0.254
7-14-1	35.618	5.869	3.077	0.242
7-25-1	28.050	4.300	2.357	0.186
7-26-1	27.451	4.187	2.285	0.180
7-27-1	27.601	4.167	2.272	0.179
**7-30-1**	26.767	3.928	2.145	0.169

### Comparative Performance of Forecast by ARIMA and ANN

**Fig. 3.**
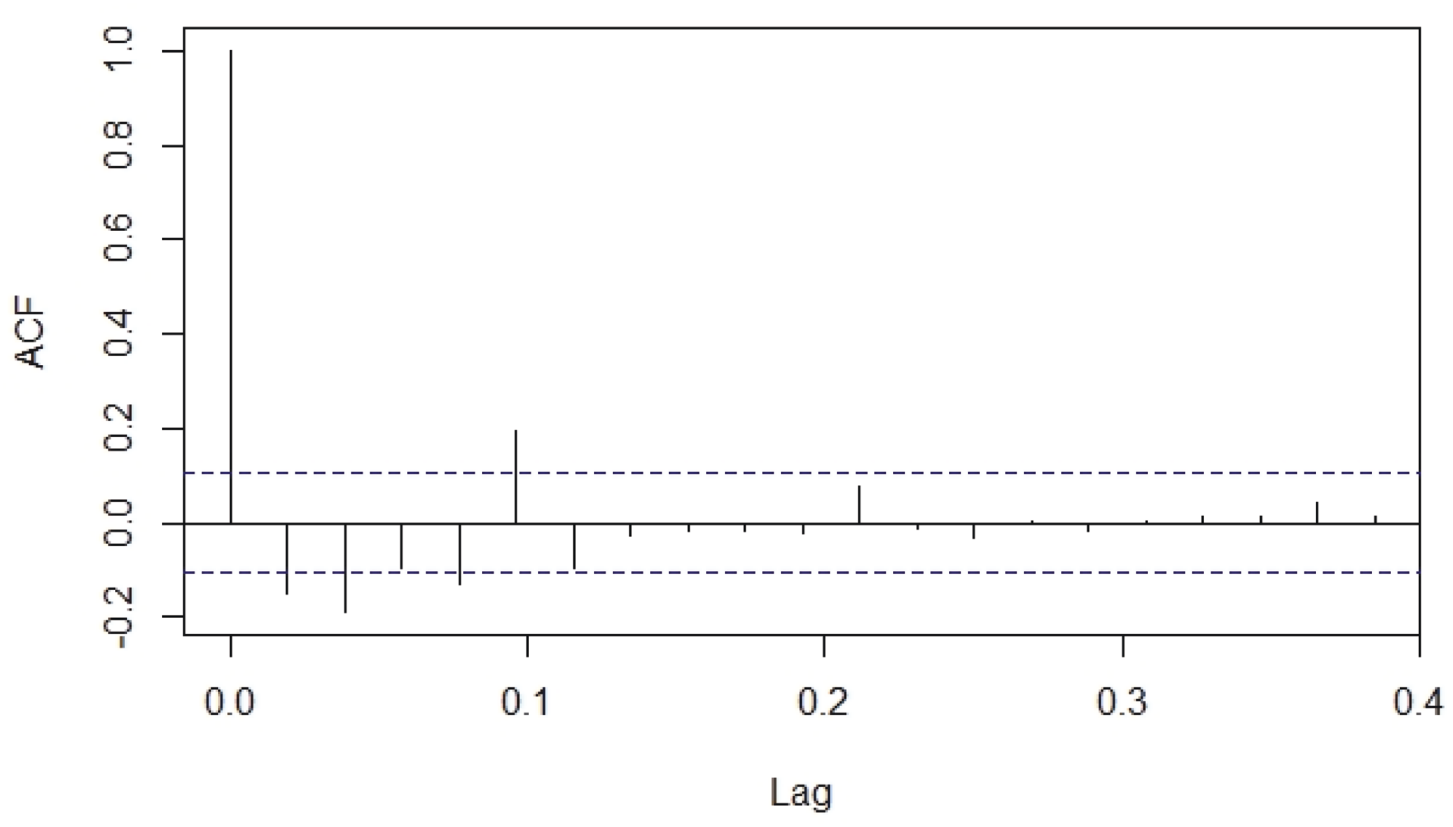
Auto Correlation Function (ACF) plot after first differentiation of the *H. armigera* trap data.

**Fig. 4.**
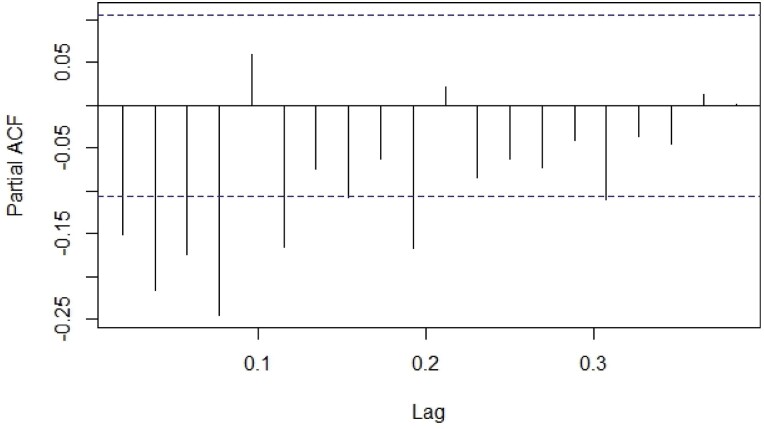
Partial Auto Correlation Function (PACF) plot after first differentiation of *H. armigera* trap data.

**Fig. 5.**
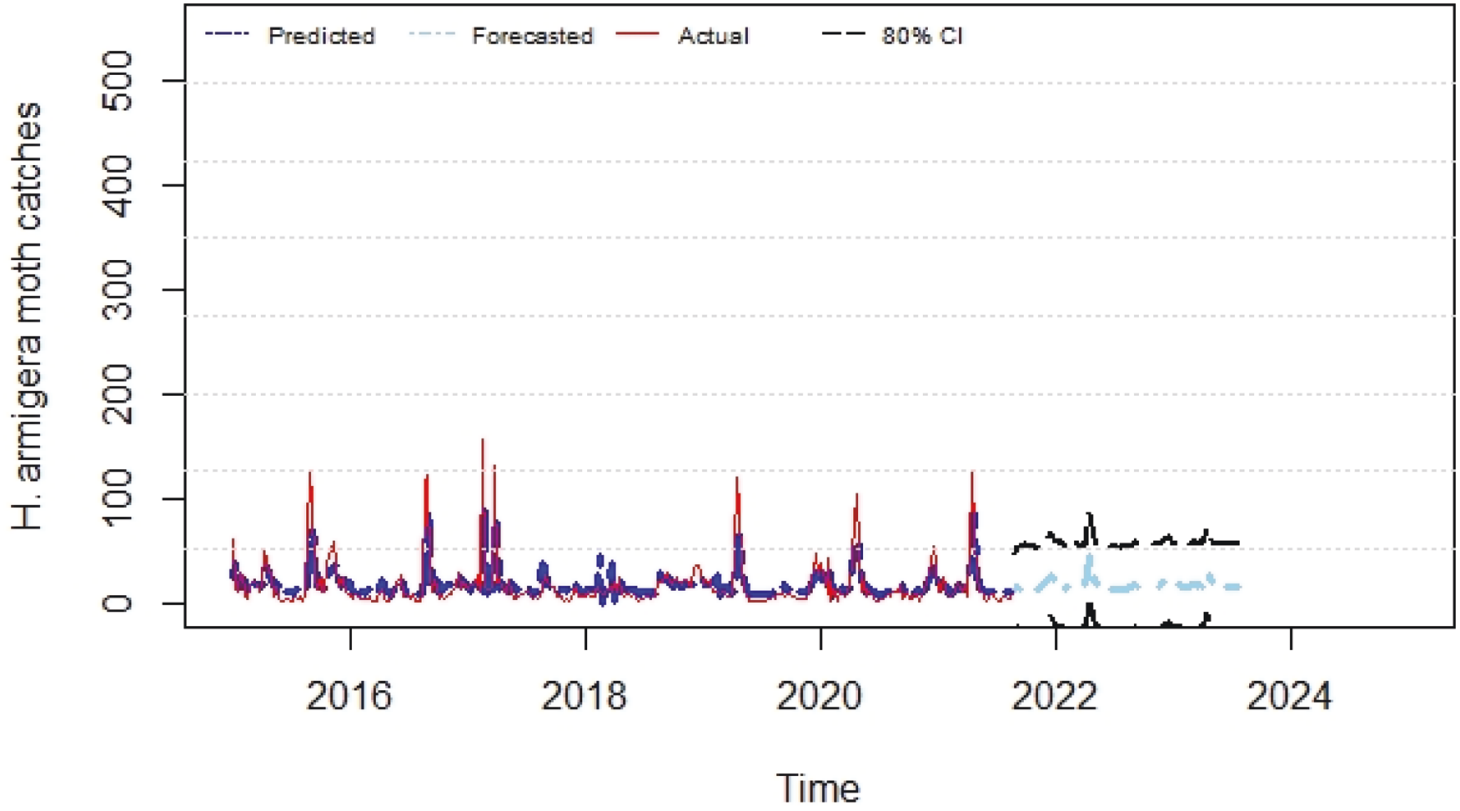
Comparison of actual *versus* fitted values of ARIMA for *H. armigera* moth catches.

**Fig. 6.**
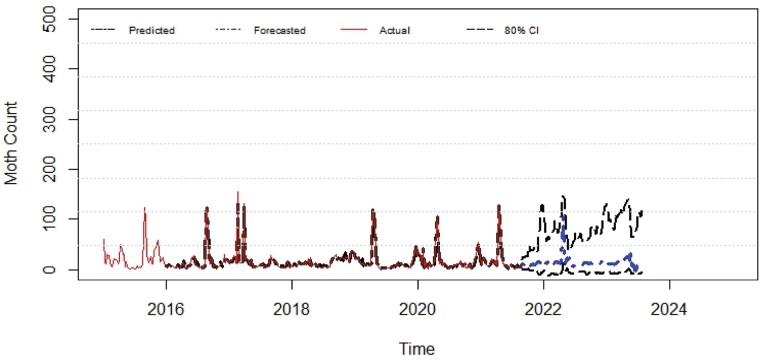
Comparison of actual versus fitted values of ANN for *H. armigera* male moth trap catches.

The predicted values obtained through ANN and ARIMA models were compared to the actual moth catches of pod borer. Comparative performance of fitted models was examined through computing RMSE, MAE, MAPE, and MASE criterion. The tenable models were identified from the developed ACF and PACF (Figs. 3 and 4). The best ANN and ARIMA models were fitted to predict the trap catches of *H. armigera* based on its historical trend over a period of 5 years. The results presented in Table 5 show that the ANN model reported lower values of RMSE (3.928), MAE (2.145), MAPE (26.767), and MASE (0.169) when compared with the ARIMA model. Both ex-ante and ex-post forecasts were made using the best fitted ANN and ARIMA models, and the results were compared with actual observations which revealed that there were narrow variations between the actual and predicted values (Figs. 5 and 6). The data presented in Table 6 depicts the comparison of ARIMA and ANN predicted values with actual catches of *H. armigera*. Forecasted values of *H. armigera* moth catches up to August (31 Standard Meteorological Week [SMW]), 2023 by selected best fits of ARIMA and ANN models are presented in Table 7.

**Table 5.** Comparison of ARIMA and ANN best fit model performance

Criterion	Model	
	ARIMA	ANN
MAE	9.42	2.145
MAPE	93.70	26.767
RMSE	17.74	3.928
MASE	1.02	0.169

**Table 6.** Comparison of ARIMA and ANN predicted values with actual moth catches of *H. armigera*

Year	SMW	Months	Actual moth catches	Forecasted moth catches	
				AIRIMA	ANN
2021	25	June	12	12	14
2021	26	July	9	10	12
2021	27	July	12	14	10
2021	28	July	11	13	8
2021	29	July	10	12	14
2021	30	July	7	10	8
2021	31	August	11	13	13
2021	32	August	9	9	12
2021	33	August	15	12	16
2021	34	August	19	26	12
2021	35	September	–	23	35
2021	36	September	–	33	50
2021	37	September	–	38	56
2021	38	September	–	43	92
2021	39	September	–	37	49
2021	40	October	–	36	54
2021	41	October	–	66	56
2021	42	October	–	43	57
2021	43	October	–	22	57
2021	44	November	–	40	58
2021	45	November	–	36	58
2021	46	November	–	24	57
2021	47	November	–	32	55
2021	48	December	–	37	56
2021	49	December	–	63	54
2021	51	December	–	21	51
2021	52	December	–	11	48

SMW, Standard metrological week.

**Table 7.** Forecasted values of *H. armigera* moth catches by selected best fits of ARIMA and ANN model

Year	SMW	Months	Actual moth catches	Forecasted moth catches	
				ARIMA	ANN
2022	1	January	–	6	42
2022	2	January	–	12	39
2022	3	January	–	11	37
2022	4	January	–	11	35
2022	5	February	–	13	34
2022	6	February	–	11	34
2022	7	February	–	11	32
2022	8	February	–	13	32
2022	9	March	–	12	33
2022	10	March	–	12	36
2022	11	March	–	12	38
2022	12	March	–	15	39
2022	13	March	–	15	39
2022	14	April	–	13	40
2022	15	April	–	14	45
2022	16	April	–	25	46
2022	17	April	–	25	47
2022	18	May	–	31	48
2022	19	May	–	36	47
2022	20	May	–	37	48
2022	21	May	–	31	48
2022	22	June	–	20	37
2022	23	June	–	17	26
2022	24	June	–	16	24
2022	25	June	–	20	25
2022	26	July	–	21	23
2022	27	July	–	16	27
2022	28	July	–	24	21
2022	29	July	–	23	19
2022	30	July	–	26	28
2022	31	August	–	20	25
2022	32	August	–	19	24
2022	33	August	–	26	9
2022	34	August	–	35	51
2022	35	September	–	54	84
2022	36	September	–	56	91
2022	37	September	–	57	78
2022	38	September	–	58	112
2022	39	September	–	45	79
2022	40	October	–	39	78
2022	41	October	–	47	75
2022	42	October	–	46	77
2022	43	October	–	51	71
2022	44	November	–	53	71
2022	45	November	–	55	66
2022	46	November	–	53	46
2022	47	November	–	48	53
2022	48	December	–	45	56
2022	49	December	–	42	72
2022	50	December	–	39	50
2022	51	December	–	36	44
2022	52	December	–	32	33
2023	1	January	–	27	20
2023	2	January	–	23	24
2023	3	January	–	19	63
2023	4	January	–	12	17
2023	5	January	–	9	13
2023	6	February	–	9	10
2023	7	February	–	10	12
2023	8	February	–	9	12
2023	9	February	–	9	14
2023	10	March	–	7	15
2023	11	March	–	13	14
2023	12	March	–	17	16
2023	13	March	–	10	18
2023	14	April	–	7	21
2023	15	April	–	12	22
2023	16	April	–	9	22
2023	17	April	–	11	23
2023	18	May	–	11	25
2023	19	May	–	9	27
2023	20	May	–	17	28
2023	21	May	–	10	29
2023	22	May	–	23	29
2023	23	June	–	20	30
2023	24	June	–	15	32
2023	25	June	–	28	33
2023	26	June	–	18	34
2023	27	July	–	9	35
2023	28	July	–	15	36
2023	29	July	–	16	38
2023	30	July	–	31	39

SMW, Standard meteorological week.

Based on ARIMA and ANN, predicted *H. armigera* population trap catches were low during the rainy season, moderate during post rainy season, and high in months of rabi season. The ARIMA results predicted that *H. armigera* male adult population would be persistent throughout the year with huge week-to-week variations and adult trap catches would be higher from September 2021 (35 SMW) to May 2022 (20 SMW), with high chances of incidence likely to occur in early sowing legume crops like chickpea and pigeonpea. It also predicted a sharp decline in the *H. armigera* population during June, July, and August months of the years 2022 and 2023 (21–33 SMW), then a steady increase from September, 2022 (35 SMW) and the moth activity prevailed till July 2023 (30 SMW).

## Discussion

Our results demonstrate that both ARIMA and ANN forecasted results are more proximal to the original historical trap data in performing forecast modelling for pod borer over the next two-three years. The ARIMA modelling has been employed by many researchers to predict incidence of pest populations. In our current research, predicted a fall in the *H. armigera* population during the months of June, July, and August in the years 2022 and 2023 (21–33 SMW), followed by a steady increase in the beginning of September 2022 (35 SMW) and lasting until July 2023 (30 SMW). Our results corroborated with Boopathi et al. (2015) who developed a forecasting model to predict lychee bug, *T. papillosa* incidences in lychee orchards using the autoregressive integrated moving average (ARIMA) model of time-series analysis. The predicted highest *T. papillosa* incidence during April 2010, January 2011, May 2012, and February 2013. Elango et al. (2021) also used different prediction models by fitting covariates to the time series data and concluded that ARIMA (0,2,1) model with maximum temperature was best for predicting the rugose spiralling whitefly (*Aleurodicus rugioperculatus*) incidence. Similarly, the ANN was employed by Gupta et al. (2003), Patil and Mythri (2013), and Kumari et al. (2013) to predict the population dynamics of cotton thrips, *Thrips tabaci* (Lindae), and forecasting of pod damage by *H. armigera* with Multi-Layer Perceptron (MLP) neural network structure with Backpropagation training algorithm. With the addition of weather parameters as exogeneous variables ARIMAX models can be developed to assess the influence of weather on insect pest incidence and distribution. In a study of factors contributing to increase in incidence of greenhouse whitefly (*Trialeurodes vaporariorum*), Chiu et al. (2019) used ARIMA and ARIMAX models to forecast its incidence and found that temperature and humidity were the key contributing exogeneous factors increased abundance in green houses. Most of the previously developed prediction models were based on linear regression and mathematical equations, thus were preliminary in nature. The present methodology of using ARIMA and ANN combines both machine language and artificial network intelligence where the input information is summed up in the computing unit (artificial neuron). It is an improved prediction model with better prediction accuracy compared to other traditionally used linear models in field for predicting *H. armigera* infestation.

Despite the apparent suitability of time series models for studying the pest population dynamics of old-world bollworm, *H. armigera*, these models have not been widely used to describe the temporal and spatial dynamics of insect pests. This study appears to be the first of its kind where in a time series model has been used to describe the temporal dynamics of *H. armigera* in field crops in India. Several researchers have used ARIMA, ANN, and ARIMAX models to forecast the future disease occurrence (Souza et al. 2015), stock price forecasting (Adebiyi et al. 2014), and crop yield predictions (Rathod et al. 2017). In this study we presented an intelligent system by comparing ARIMA and ANN for effectual prediction of pest population dynamics of *H*. *armigera*. Based on the results of current study we can clearly mark out the months with number of trap catches, which would be useful in formulating the timely pest control measures.

## Conclusions

Insect pest forecasting is a vital component in integrated pest management. Its integration with other pest management activities makes it one of the most successful tools. The historical pheromone trap catch data would be helpful in modelling and forecasting of *H. armigera* populations. The prediction models built in this study using ANN and ARIMA will further help to predict the incidence and population surge of *H. armigera* in time which in turn would aid in taking preemptive measures for successful suppression of the pest. Among the methods used, the ANN based models outperformed the ARIMA model based on four different performance metrics. Results demonstrate that the ANN based model can forecast pod borer moth catches closely to the actual moth incidences with 80% accuracy. The results obtained proved that the model (ANN:7-30-1) can be used for forecasting the future trend and occurrence of the pod borer, *H*. *armigera*.
